# Metal Oxide Nanostructures and Their Gas Sensing Properties: A Review

**DOI:** 10.3390/s120302610

**Published:** 2012-02-27

**Authors:** Yu-Feng Sun, Shao-Bo Liu, Fan-Li Meng, Jin-Yun Liu, Zhen Jin, Ling-Tao Kong, Jin-Huai Liu

**Affiliations:** 1 Department of Mechanical and Automotive Engineering, Anhui Polytechnic University, Wuhu 241000, China; E-Mail: sunyufeng118@126.com; 2 Research Center for Biomimetic Functional Materials and Sensing Devices, Institute of Intelligent Machines, Chinese Academy of Sciences, Hefei 230031, China; E-Mails: jyliu@iim.ac.cn (J.-Y.L.); zjin@iim.ac.cn (Z.J.); ltkong@iim.ac.cn (L.-T.K.); jhliu@iim.ac.cn (J.-H.L.); 3 Wuhu Returned Overseas Students’ Enterprise Park, Wuhu 241000, China; E-Mail: shaoboliu@126.com

**Keywords:** metal oxide, gas sensing, nanostructure, size effect, doping

## Abstract

Metal oxide gas sensors are predominant solid-state gas detecting devices for domestic, commercial and industrial applications, which have many advantages such as low cost, easy production, and compact size. However, the performance of such sensors is significantly influenced by the morphology and structure of sensing materials, resulting in a great obstacle for gas sensors based on bulk materials or dense films to achieve highly-sensitive properties. Lots of metal oxide nanostructures have been developed to improve the gas sensing properties such as sensitivity, selectivity, response speed, and so on. Here, we provide a brief overview of metal oxide nanostructures and their gas sensing properties from the aspects of particle size, morphology and doping. When the particle size of metal oxide is close to or less than double thickness of the space-charge layer, the sensitivity of the sensor will increase remarkably, which would be called “small size effect”, yet small size of metal oxide nanoparticles will be compactly sintered together during the film coating process which is disadvantage for gas diffusion in them. In view of those reasons, nanostructures with many kinds of shapes such as porous nanotubes, porous nanospheres and so on have been investigated, that not only possessed large surface area and relatively mass reactive sites, but also formed relatively loose film structures which is an advantage for gas diffusion. Besides, doping is also an effective method to decrease particle size and improve gas sensing properties. Therefore, the gas sensing properties of metal oxide nanostructures assembled by nanoparticles are reviewed in this article. The effect of doping is also summarized and finally the perspectives of metal oxide gas sensor are given.

## 1. Introduction

The issue of air quality is still a major concern in many countries. A clean air supply is essential to our health and the environment. The human nose serves as a highly advanced sensing system which may differentiate between hundreds of smells but fails if absolute gas concentrations or odorless gases need to be detected. The demand for detecting toxic and deleterious gases is accordingly urgent to support or replace human nose. Although a large number of gas detecting systems have currently been used in process control and laboratory analytics [1–4], high performance gas sensors with high sensitivity, high selectivity and rapid response speed are also needed to improve the levels of gas detection.

Metal oxide gas sensors have been widely used in portable gas detection systems because of their advantages such as low cost, easy production, compact size and simple measuring electronics [5,6]. However, the performance of such sensors is significantly influenced by the morphology and structure of sensing materials, resulting in a great obstacle for gas sensors based on bulk materials or dense films to achieve highly-sensitive properties. Gas sensors based on nanomaterials are a greatly developing direction to improve gas sensing properties in sensitivity, selectivity and response speed. Although there are already some reviews on metal oxide gas sensor [7–9], it is still necessary to systematically summarize the features of metal oxides from the perspective of nanoscience and nanotechnology. In this review, we provide a brief summary on metal oxide nanostructures and their gas sensing properties from the aspects of particle size, morphology and doping. Most of the examples are given based on n-type metal oxides which are more extensively investigated and applied among the metal oxide gas sensors.

## 2. Gas Sensing Mechanism

It is necessary to reveal the sensing mechanism of metal oxide gas sensors which is helpful for designing and fabricating novel gas sensing materials with excellent performance. Although the exact fundamental mechanisms that cause a gas response are still controversial, it is essentially responsible for a change in conductivity that trapping of electrons at adsorbed molecules and band bending induced by these charged molecules. Herein, a brief introduction to the sensing mechanism of n-type metal oxides in air is given based on the example of SnO_2_. Typically, oxygen gases are adsorbed on the surface of the SnO_2_ sensing material in air. The adsorbed oxygen species can capture electrons from the inner of the SnO_2_ film. The negative charge trapped in these oxygen species causes a depletion layer and thus a reduced conductivity. When the sensor is exposed to reducing gases, the electrons trapped by the oxygen adsorbate will return to the SnO_2_ film, leading to a decrease in the potential barrier height and thus an increase in conductivity. There are different oxygen species including molecular (O_2_^−^) and atomic (O^−^, O^2−^) ions on the surface depending on working temperature. Generally, below 150 °C the molecular form dominates while above this temperature the atomic species are found [9,10].

**Figure 1.**
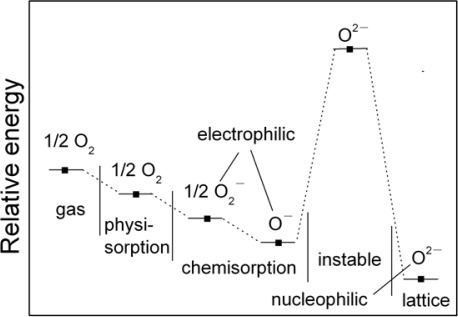
Energy diagram for various oxygen species in the gas phase adsorbed at the surface and bound within the lattice of SnO_2_. Reprinted with permission from [11]. Copyright (2007) Nova Science Publishers.

The overall surface stoichiometry has a decisive influence on the surface conductivity for the metal oxides. Oxygen vacancies act as donors, increasing the surface conductivity, whereas adsorbed oxygen ions act as surface acceptors, binding elections and diminishing the surface conductivity. Figure 1 shows the energy diagram of various oxygen species in the gas phase, adsorbed at the surface and bound within the lattice of SnO_2_ [11,12]. On SnO_2_ films the reaction O_2_^−^_ads_ + e^−^ = 2O^−^_ads_ takes place as the temperature increases. The desorption temperatures from the SnO_2_ surface are around 550 °C for O^−^_ads_ ions and around 150 °C for O_2_^−^_ads_ ions. At constant oxygen coverage, the transition causes an increase in surface charge density with corresponding variations of band bending and surface conductivity. From conductance measurements, it is concluded that the transition takes place slowly. Therefore, a rapid temperature change on the part of the sensors is usually followed by a gradual and continuous change in the conductance. The oxygen coverage adjusts to a new equilibrium and the adsorbed oxygen is converted into another species which may be used in measurement method of dynamic modulated temperature as reported previously [13–19].

## 3. Device Structure

**Figure 2.**
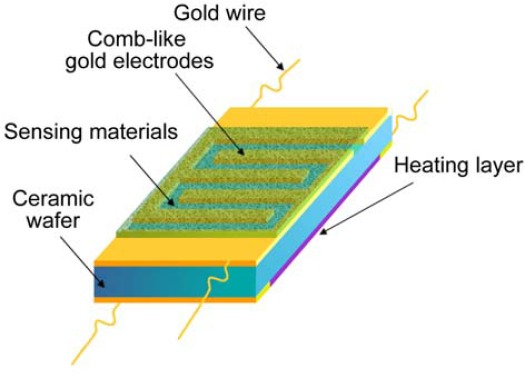
Device structure based on ceramic wafer substrate.

**Figure 3.**
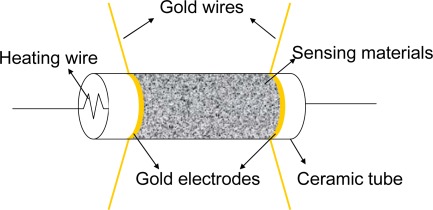
Device structure based on ceramic tube substrate.

Gas sensors based on metal oxide nanostructures generally consist of three parts, *i.e.*, sensing film, electrodes and heater. Metal oxide nanostructures react in the form of a film which will change in resistance upon exposure to target gases. A pair of electrodes is used to measure the resistance of the sensing film. Usually the gas sensors are furnished with a heater so that they are heated externally to reach an optimum working temperature. Currently, metal oxide nanostructures sensors have been characterized in three ways: conductometric, field effect transistor (FET) and impedometric ones [20]. FET type is usually exploited to fabricate sensors based on single or arrays of one-dimensional (1D) semiconducting nanomaterials, which have a complex fabrication process. Impedometric type sensors are based on impedance changes and are operated under alternating voltage upon exposure to target species, which has not yet attracted much attention. The conductometric type is the most common gas sensor which is suitable for most nanomaterials. There are two types of device structures in conductometric sensors: directly heated and indirectly heated. A directly heated type structure means the heater is contacted with the sensing material, which may lack stability and anti-interference ability, so most of the current nanostructure-based gas sensors are indirectly heated type structures which can be divided into two types, *i.e.*, cylindrical and planar layouts, as shown in Figures 2 and 3. Alumina ceramics (wafers or tubes) are generally used as substrates to support sensing films. In the ceramic tube-based device, a piece of heating wire is placed in the interior of the ceramic tube, while, in the ceramic wafer-based device, heating paste is placed on the backside of the ceramic wafer. Some silica wafers can also be used as the substrate, which is advantageous in manufacturing small sized gas sensor because of its compatibility with integrated circuits.

## 4. Nano Effect of Small Size of Metal Oxide Nanoparticles

**Figure 4.**
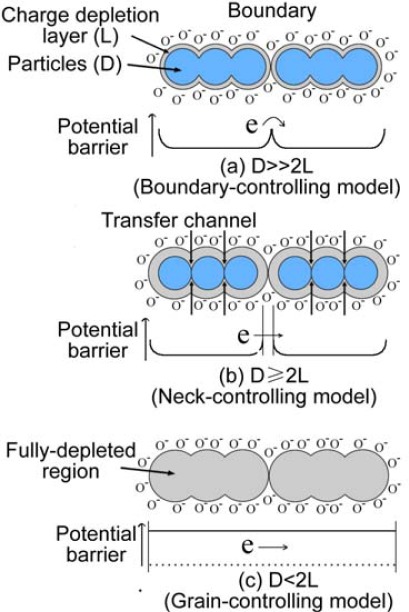
Schematic model of the effect of the crystallite size on the sensitivity of metal-oxide gas sensors: (**a**) D >> 2L; (**b**) D ≥ 2L; (**c**) D < 2L.

The “small size effect” of metal oxides has been reported by many publications [21–27]. As shown in Figure 4, a sensor is considered to be composed of partially sintered crystallites that are connected to their neighbors by necks. Those interconnected grains form larger aggregates that are connected to their neighbors by grain boundaries [28]. On the surface of the grains, adsorbed oxygen molecules extract electrons from the conduction band and trap the electrons at the surface in the form of ions, which produces a band bending and an electron depleted region called the space-charge layer. When the particle size of the sensing film is close to or less than double the thickness of the space-charge layer, the sensitivity of the sensor will increase remarkably. Xu *et al.* explained the phenomena by a semiquantitative model [29]. Three different cases can be distinguished according to the relationship between the particle size (D) and the width of the space-charge layer (L) that is produced around the surface of the crystallites due to chemisorbed ions and the size of L is about 3 nm for pure SnO_2_ material in literatures [30–34]. When D >> 2L, the conductivity of the whole structure depends on the inner mobile charge carriers and the electrical conductivity depends exponentially on the barrier height. It is not so sensitive to the charges acquired from surface reactions. When D ≥ 2L, the space-charge layer region around each neck forms a constricted conduction channel within each aggregate. Consequently, the conductivity not only depends on the particle boundaries barriers, but also on the cross section area of those channels and so it is sensitive to reaction charges. Therefore, the particles are sensitive to the ambient gas composition. When D < 2L, the space-charge layer region dominates the whole particle and the crystallites are almost fully depleted of mobile charge carriers. The energy bands are nearly flat throughout the whole structure of the interconnected grains and there are no significant barriers for intercrystallite charge transport and then the conductivity is essentially controlled by the intercrystallite conductivity. Few charges acquired from surface reactions will cause large changes of conductivity of the whole structure, so the crystalline SnO_2_ becomes highly sensitive to ambient gas molecules when its particle size is small enough.

**Figure 5.**
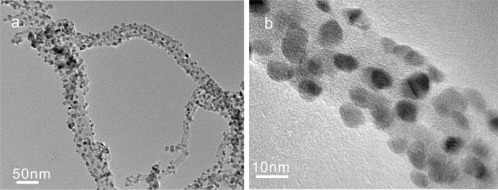
(**a**) Low- and (**b**) high-magnified TEM images of the SnO_2_/MWCNT nanocomposites. Reprinted with permission from [38]. Copyright (2010) RSC Publishing.

Based on Xu’s model, many new sensing materials are developed to achieve high gas sensing properties [35–37]. Typically, the nanocomposite of SnO_2_ and multiwall carbon nanotube (MWCNT) was exploited to detect persistent organic pollutants (POPs) which possess stable chemical properties and are ordinarily difficult to detected with metal oxides [38]. The preparation of materials with size and porosity in the nanometer range is of technological importance for a wide range of sensing applications. The ultrasensitive detection of aldrin and dichlorodiphenyltrichloroethane (DDT), has been carried out using the nanocomposite of small SnO_2_ particles and MWCNTs. The nanocomposite shows a very attractive improved sensitivity compared with a conventional SnO_2_ sensor. A sharp response of low limiting concentration about 1 ng was observed in both aldrin and DDT, suggesting potential applications as a new analytical approach. One major advantage of this sensing material is its stable attachment between sub-10 nm SnO_2_ nanoparticles and carbon nanotubes shown is Figure 5. Besides, the SnO_2_/MWCNT nanocomposite synthesized by a wet chemical method may control the size of SnO_2_ particles under 10 nm and form highly porous three dimensional (3D) structures. Among the highly porous 3D structures, MWCNTs can be regarded as the framework and the SnO_2_ particles uniformly packed on them, which may enhance the ability of gas diffusion into and out of the sensing film. The high sensitivity can also be attributed to an effect of p-n junction formed between p-type carbon nanotubes and n-type SnO_2_ nanoparticles. The investigation results make SnO_2_/MWCNT nanocomposites attractive for the purpose of POPs detection.

## 5. Porous Film of Metal Oxides

Commonly, metal oxide sensing films are divided into dense and porous [10]. In dense films, the gas interaction takes place only at the surface of the film since the analyte cannot penetrate into the sensing film. In porous films, the gas can penetrate into the film and interact with the inner grains. In fact, metal oxide films are usually produced with a certain overall porosity through several processes, which is yet insufficient for gas sensing.

**Figure 6.**
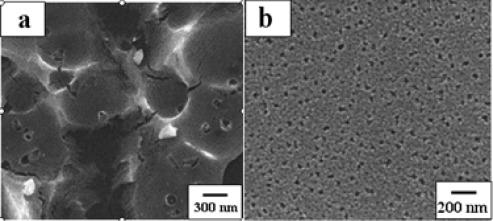
(**a**) Low- and (**b**) high-magnified SEM images of the mesoporous SnO_2_. Reprinted with permission from [57]. Copyright (2010) Elsevier Ltd.

Apart from large surface-to-volume ratios, well-defined and uniform pore structures are particularly desired for metal oxides to improve sensing performance. Porous materials are classified into several kinds according to their size. According to the definition of the International Union of Pure and Applied Chemistry (IUPAC) [39], microporous materials have pore diameters of less than 2 nm and macroporous materials have pore diameters of greater than 50 nm; the mesoporous category thus lies in the middle. Mesoporous materials have lots of application in the fields of drug delivery, catalysts, energy storage and detection of gas pollution. Mesoporous oxide structures with well-aligned pore structures are fascinating for gas sensing investigation. For example, mesoporous SnO_2_ has attracted more interests because of their high sensitive and rapid gas response, which can facilitate gas diffusion and mass transport due to mesopores providing regions for exchanging gases. Mesoporous materials can be prepared via many methods such as template synthesis [40–43], hydrothermal/solvothermal approaches [44–46], self-assembly reaction [47–51], the Kirkendall effect [52–54], Ostwald ripening [55,56] and so on. Among those methods, the mesoporous SnO_2_ (Figure 6) prepared by the method of MWCNT templates exhibited an excellent gas sensing properties [57].

Compared to traditional SnO_2_, the mesoporous SnO_2_ has better permeability because mesoporous SnO_2_ provides more space for gas molecules to diffuse in and out of the film. The gas sensing comparison indicates SnO_2_ mesoporous materials have much better response to ethanol and benzene, especially benzene. The key parameters to determine the gas sensing characteristics are thickness, permeability and surface morphology, while mesoporous structure has better permeability. During the response and recovery process, target gas molecules diffuse in and out of SnO_2_ film. A diffusion equation assuming a first-order reaction of target gas is inducted by Sakai and co-workers to explain gas diffusion dynamics in the response process. In the mesopores, gas diffusion constant (D_k_) is determined by temperature (T), pore radius (r), molecular weight (M) of the diffusion gas as following equation [58]:

(1) $$D_{k}=\frac{4r}{3}\;\sqrt{\frac{2\mathrm{RT}}{\pi M}}$$

where R is gas constant. The molecules of sample gas diffuse into the surface of the mesoporous SnO_2_ film and react with the surface oxygen of SnO_2_ chains subsequently [58,59]. The reaction of molecules occurs only on the out surface region of the traditional SnO_2_ film. Since the SnO_2_ mesoporous structure can increase response region and the inner parts become active, the mesoporous SnO_2_ materials are more sensitive.

## 6. Porous Nanostructures of Metal Oxide and Their Gas Sensing Properties

In the past few years, many efforts have been devoted to improve the sensitivity of gas sensors. Sakai *et al.* found that the porous structure of the sensing film played a critical role in the performance of the sensor because it decided the rate of gas diffusion [58]. Xu *et al.* found that the particle size heavily affected the sensitivity of sensor [29]. Although many methods have been reported to synthesize monodisperse nanoparticles of metal oxides [60–64], small size of nanoparticles are not stable which may easily congregate and grow up under heating conditions [31]. Besides, small sized nanoparticles will be compactly sintered together during the film coating process which is a disadvantage for gas diffusion in them. If porous nanostructures are used as gas sensing materials, the gas sensing properties will be much improved. On the basis of those reasons, nanoparticles-assembled nanostructures with many kinds of shapes such as porous nanowires, porous nanotubes, porous nanospheres and so on are reviewed in this chapter, which exhibited excellent gas sensing properties because they not only possessed large surface area and relatively mass reactive sites, but also formed relatively loose film structures.

### 6.1. Porous Nanowires

One-dimensional or quasi-1D metal oxide nanostructures possess very large surface-to-volume ratios which is advantageous in gas sensing. Besides, other factors also make these nanostructures particularly suitable for conductimetric gas sensing as follows: (i) the comparability of the Debye screening length of nanostructured metal oxides with their lateral dimensions and (ii) the ability to fabricate them routinely with significant lengths providing a long semiconducting channel. All these make 1D or quasi-1D nanostructures such as nanowires, nanotubes and nanorods highly sensitive and efficient transducers of surface chemical processes into electrical signals [65].

**Figure 7.**
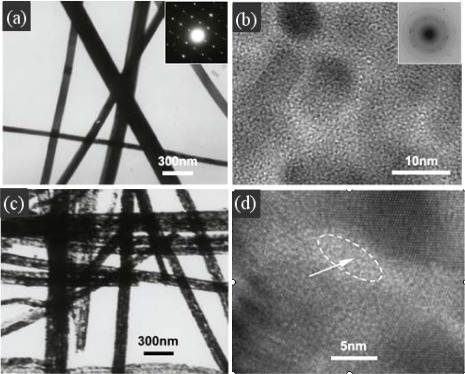
(**a**) TEM images of the precursor nanowires, (**b**) HRTEM image taken on the single precursor nanowire, (**c**) TEM and (**d**) HRTEM image of highly porous CdO nanowires. Reprinted with permission from [80]. Copyright (2008) IOP Publishing Ltd.

Nanowires as a kind of important one-dimensional nanostructures have been used in many field [66,67]. Many kinds of semiconductor nanowires, such as SnO_2_ [68–70], In_2_O_3_ [71,72], ZnO [73–75], TiO_2_ [76,77] and so on, have been widely applied in gas sensors. However, smooth nanowires only adsorb gases at their surfaces which results in a great obstacle to achieve highly-sensitive properties. Porous nanowires have attracted great interests due to their high surface-to-volume ratio and porous structure which allows adsorbing gases not only on the surface but also throughout the bulk. Wang *et al.* [78,79] have prepared porous SnO_2_ nanowires based on glycolate precursors under mild conditions which showed good sensitivity to some gases such as C_2_H_5_OH, CO and H_2_. Guo *et al.* have prepared highly porous CdO nanowires as shown in Figure 7 by calcining the hydroxy- and carbonate-containing cadmium compound precursor nanowires [80]. The precursor converted into porous CdO nanowires, which were polycrystalline structure, through heat treatment in air without changing the wire-like topography. Due to the highly porous structure, the highly porous CdO nanowires showed rapid response, low detection limit, high signal-to-noise ratio and selectivity to nitrogen oxide which is one of the most dangerous air pollutants.

### 6.2. Porous Nanotubes

**Figure 8.**
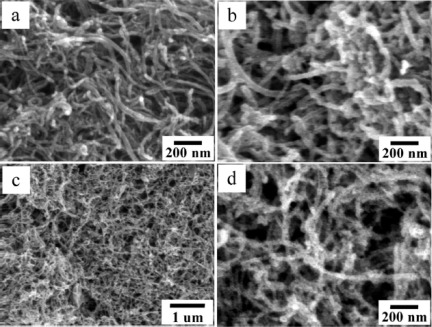
(**a**) FESEM images of the purified MWCNTs, (**b**) SnO_2_/MWCNT nanocomposites and (**c**,**d**) porous SnO_2_ nanotubes. Reprinted with permission from [88]. Copyright (2009) American Chemical Society.

Nanotubes are also one kind of widely-used one-dimensional nanostructures. Because of their hollow structure, nanotubes possess higher porosity and larger surface area than nanowires [81]. So, it is more favorable for gas sensor to use nanotubes as gas sensing materials instead of nanowires. However, the preparation process is more complicated for nanotubes. Metal oxide nanotubes were mainly prepared through hydrothermal synthesis [82,83], anodizing processes [84,85] or templated sol-gel processes [86]. For example, Wang *et al.* prepared SnO_2_ nanotubes by a sol-gel template (anodic aluminium oxide template) synthetic technique [86]. SnO_2_ sol was forced to pass through the pores of the anodic aluminium oxide template and adhere on the pore walls. Then, SnO_2_ tubes formed after annealing treatment. Although the SnO_2_ nanotubes exhibited an enhanced sensitivity towards ethanol gas than SnO_2_ nanopowders, the synthesis process is complicated and the production yields are also limited. So, bulk production of metal oxide nanotubes with excellent gas sensing properties is desired for the researchers. It is reported that MWCNTs were used as templates to fabricate SnO_2_ nanotubes by a wet-chemical method [87]. Furtherly, Jia *et al.* have prepared SnO_2_ nanotubes by using MWCNTs as templates [88] as shown in Figure 8, which is more porous than the one prepared by a sol-gel template method. Besides, the SnO_2_ crystallite size is about 5 to 7 nm, a size ideal for gas sensing, so the porous SnO_2_ nanotubes exhibited an excellent response and reversibility to some organic gases, such as ethanol and acetone, of which the responses (defined as R_a_/R_g_, where, R_a_ is the resistance in air and R_g_ is that in the mixture of air and target gases) to 100 ppm ethanol and acetone were as high as 130 and 126, respectively.

### 6.3. Porous Nanosheets

**Figure 9.**
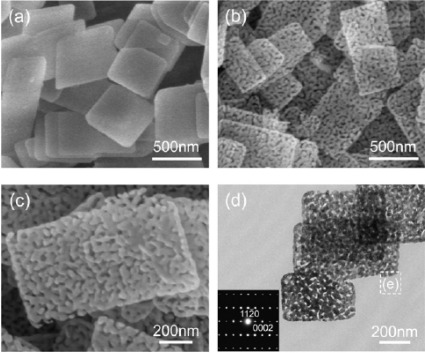
(**a**) FESEM images of the as-synthesized precursor, (**b**) porous ZnO nanosheets, (**c**) their high-magnification observation, (**d**) low-magnification image with the corresponding SAED pattern as an inset. Reprinted with permission from [92]. Copyright (2009) IOP Publishing Ltd.

Previous work has indicated that polycrystalline structural sensing materials have high response and poor stability. In contrast, single-crystalline materials exhibit low response and good stability. For sensor developers, the difficulty is to maintain the balance of high response and good stability. Porous metal oxide nanosheets have the characteristic of remaining a single-crystalline structure and providing a relatively high surface area [89,90]. Therefore, porous single-crystalline nanostructures are the ideal material which maintains a balance between high response and good stability. Sysoev *et al.* have investigated the gas sensing properties of single-crystalline SnO_2_ nanowires which revealed high sensitivity and long-term stability [91]. However, it is difficult to synthesize a large amount of porous single-crystalline nanowires. For nanosheets, it is relatively easy to simultaneously possess single-crystalline structure and lots of pores. Liu *et al.* have prepared novel single-crystalline ZnO nanosheets with porous structure via annealing ZnS(en)_0.5_ (en = ethylenediamine) complex precursor as shown in Figure 9 [92]. There are numerous mesopores with a diameter of about 26.1 nm all through each nanosheet in a high density. Besides, ZnO nanosheets gas sensor not only exhibits good response and short response and recovery time, but also have stability in a long term. The research results confirm that it is feasible to fabricate highly sensitive and stable gas sensors based on porous single-crystalline nanomaterials. Besides, similar two dimensional porous nanostructures also exhibited excellent gas sensing properties [93–96].

### 6.4. Hollow and Porous Nanospheres

**Figure 10.**
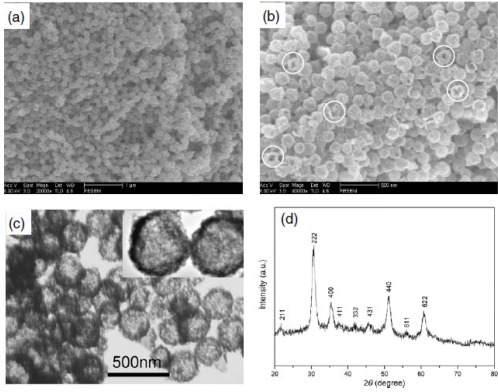
(**a**) Low magnification SEM, (**b**) high magnification SEM, (**c**) TEM images and insert image is the high magnification, (**d**) XRD pattern of the as-obtained hollow In_2_O_3_ nanospheres. Reprinted with permission from [101]. Copyright (2008) IOP Publishing Ltd.

Hollow and porous oxide structures have advantages for gas sensing application since the structures are favorable for gas diffusion [97,98]. Therefore, hollow and porous nanospheres have been widely-used in gas sensors which may adsorb gases both on the outer- and inner-surfaces [99,100]. Guo *et al.* have prepared In_2_O_3_ hollow and porous nanospheres as shown in Figure 10 by the hydrolysis of InCl_3_ using carbonaceous spheres as templates following heat treatment [101].

The In_2_O_3_ nanospheres obtained have a uniform diameter of around 200 nm and hollow structures with thin shells of about 30 nm. It is just the hollow and porous structure that In_2_O_3_ nanospheres have much larger surface area, so the In_2_O_3_ nanospheres exhibit a good response and reversibility to volatile organic compounds such as methanol, alcohol, acetone and ethyl ether. Wang *et al.* also prepared hollow SnO_2_ nanospheres by carbonaceous spheres as templates which also showed high sensitivity to triethylamine and ethanol [102].

## 7. Doping of the Metal Oxide Nanostructures in Nanoscale Levels

**Figure 11.**
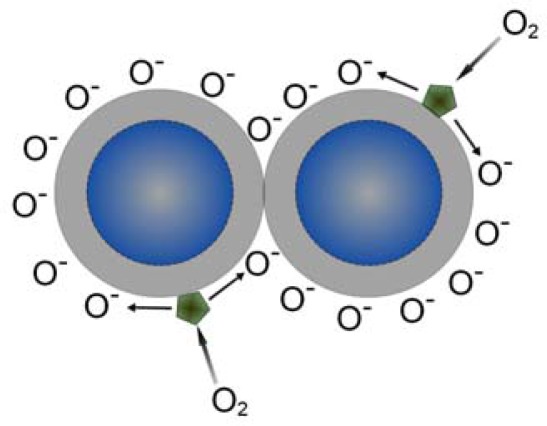
Oxygen spillover process in the surface of doped metal oxides.

Doping of metal oxide sensing film is a traditional technology for gas sensors. The traditional concept of doping is to enhance catalytic activity and adjust electrical resistance of the intrinsic metal oxide [103–105]. The dopant is usually high active, which make it react preferentially with adsorbed molecules. As shown in Figure 11, the dopant is generally dispersed on the metal oxide matrix so that they are available near all the intergranular contacts. In air, the oxygen molecules react preferentially with the dopant forming oxygen anions and then spill over to the metal oxide matrix. When the target gases are adsorbed on to the surface of the dopant and then migrate to the oxide surface to react with surface oxygen species thereby increasing the surface conductivity [106].

However, as the development of nanotechnology, doping is given many novel meanings. A typical doping phenomenon concerns the fact that the particle size of the doped metal oxide becomes smaller than the pure one [27,28] which can be explained by Nae-Lih Wu’s theory [29], *i.e.*, because of the interaction on the boundaries between host and dopant crystallites, the motion of crystallites is restricted [107–109]. In other words, the advancing of grain boundaries which is required for crystal growth is stunted. As a result, the size of crystallites is decreased by the doping of impurities.

Gong *et al.* have investigated the role of the Cu doping in enhancing the capability to adsorb CO molecules [110]. According to their results, the Cu site in ZnO film plays an important role to adsorb CO molecules at both low and high temperatures. When CO molecules are adsorbed on the film, they are preferably adsorbed on the Cu sites to form bonds between Cu and CO. The interacting bonding between Cu and CO consists of the donation of CO 5σ electrons to the metal and the back donation of π electrons from d-orbitals of Cu to CO. That adsorption results in the enhancement of the reactivity to CO. The CO adsorption mainly takes place at the Cu sites but not at the Zn sites, and then CO molecules migrate from the Cu to the Zn sites [111], by which the Cu sites enhance the CO adsorption and thus the reaction of CO with oxygen species.

Another important role of doping in enhancing gas sensing properties is to form p-n junctions which may increase the depletion barrier height due to the electron transfer from n-type materials to p-type ones [112]. When the sensor was exposed to reducing target gases, the electrons trapped by absorbed oxygen species and p-type materials are feed back to n-type materials through surface interactions, resulting in a significantly decreased sensor resistance. Therefore, the sensor response was improved remarkably.

**Figure 12.**
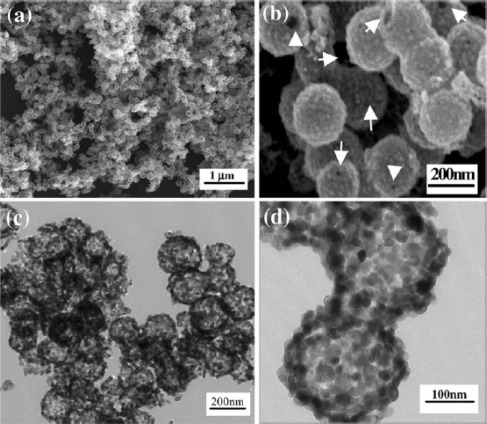
(**a**) and (**b**) FESEM images of CuO-doped SnO_2_ hollow spheres, (**c**) and (**d**) TEM images of CuO-doped SnO_2_ hollow spheres. Reprinted with permission from [116]. Copyright (2009) Springer.

**Figure 13.**
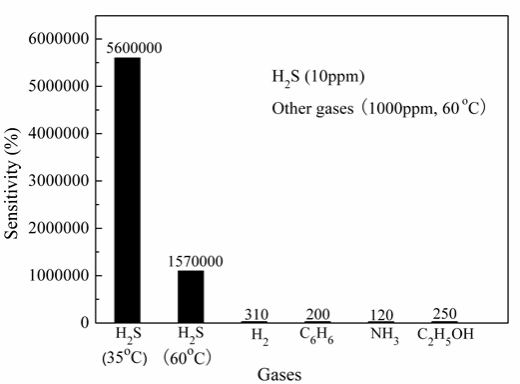
Selectivity for H_2_S gas from gas mixtures. Reprinted with permission from [116]. Copyright (2009) Springer.

If the doping is integrated into a high-sensitive nanostructure, the sensitivity will be further improved. Xue *et al.* have prepared n-type SnO_2_ nanorods uniformly coated with p-type CuO nanoparticles via a hydrothermal method which exhibited super-high sensitivity to H_2_S [113]. Besides, both the gold- or Pt-doped In_2_O_3_ nanowires have revealed higher sensitivity than the bare ones [114,115]. He *et al.* further improved the sensing properties to H_2_S of CuO-doped SnO_2_ material by replacing SnO_2_ nanorods with SnO_2_ hollow spheres. The CuO-doped SnO_2_ hollow spheres as shown in Figure 12 exhibited a ppb-leveled detection limit at a relatively low working temperature of 35 °C [116]. Besides, high selectivity was also acquired as shown in Figure 13, from which it can be seen that CuO-doped SnO_2_ hollow spheres could distinguish a small amount of (10 ppm) H_2_S among large amount of other gases including 1000 ppm of H_2_, NH_3_, ethanol and benzene.

**Figure 14.**
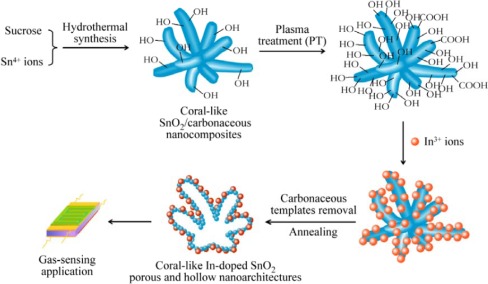
Schematic illustration of the plasma-assisted strategy for preparing highly dense In-doped SnO_2_ coral-like nanostructures for gas-sensing applications. Reprinted with permission from [120]. Copyright (2011) IOP Publishing Ltd.

Recently, metal oxide nanosturctures has been doped by many physical or chemical methods, such as thermal evaporation [117], sputter deposition [118], spin coating [119] and wet chemical methods [116]. However, a new technology for uniform and dense doping is highly desired. Liu *et al.* have developed a plasma-assisted strategy for highly dense doping of metal oxide nanostructures [120]. Figure 14 has schematically illustrated the plasma-assisted strategy for preparing highly dense In-doped SnO_2_ coral-like nanostructures.

Firstly, coral-like SnO_2_/carbonaceous nanocomposites were synthesized via a hydrothermal route. Then, the nanocomposites were functionalized by plasma treatment. The densities of some functional groups, such as hydroxyl and carboxyl, can be greatly increased on the surface of nanocomposites, which is significant for further adsorbing In^3+^ ions to achieve dense doping. The plasma-treated SnO_2_/carbonaceous nanocomposites were ultrasonically dispersed in In^3+^ ion solution and left static for a long time and subsequently washed and centrifugated. Finally, the In-doped SnO_2_ coral-like nanostructures combined with porous and hollow structures were prepared by following an annealing process to remove the sacrificed carbonaceous templates. In gas-sensing measurements, the In-doped SnO_2_ coral-like nanostructures with plasma treatment exhibited highly sensitive to chlorobenzene with a high response and short response and recovery times.

## 8. Conclusions and Perspectives

Although metal oxide gas sensors are predominantly solid-state gas detecting devices with many advantages such low cost, easy production, and compact size, and thus have been widely-used in many fields such as public safety, pollutant monitoring and so on, there is still room to improve the gas sensing performance of such sensors by controlling the morphology and structure of sensing materials. Here, gas sensing mechanisms have been reviewed first for better understanding their working principles. Then, the influences of size effect, porous nanostructure and doping on nanoscale levels have been described. By considering those influencing factors on nanoscale, novel metal oxide nanostructures will be developed and then gas sensing properties of metal oxides will be much improved.

On the basis of current progress in the field of metal oxide gas sensors, it is anticipated that the following aspects would be promising directions for developing in the future: (1) novel nanostuctures or nanocomposites which may achieve super-sensitive detection; (2) combining porous nanostructures which possess fast responses and recovery characteristics to a chromatographic technique; (3) exploiting first principles to further investigate the gas sensing mechanisms. The research on gas sensors is related to many fields such as physics, chemistry, electronics and mathematics. Addressing those problems will be one of the great challenges and it is important to enhance interdisciplinary collaboration.
